# Marked response to nab-paclitaxel in EGFR mutated lung neuroendocrine carcinoma: A case report

**DOI:** 10.1097/MD.0000000000006985

**Published:** 2017-05-26

**Authors:** Jin-Yan Liang, Fan Tong, Fei-Fei Gu, Yang-Yang Liu, Yu-Lan Zeng, Xiao-Hua Hong, Kai Zhang, Li Liu

**Affiliations:** Cancer Center, Union Hospital, Tongji Medical College, Huazhong University of Science and Technology, Wuhan, China.

**Keywords:** epidermal growth factor receptor, lung neuroendocrine carcinoma, nab-paclitaxel, tyrosine kinase inhibitor

## Abstract

**Rationale:**

Lung cancer is the leading cause of cancer-related death in the world. Tyrosine kinase inhibitors (TKIs), which target mutated epidermal growth factor receptor (EGFR), have been the first-line treatment of late-stage lung adenocarcinoma harboring EGFR mutation. EGFR mutations are mostly identified in lung adenocarcinoma. However, it is rarely seen in lung neuroendocrine carcinoma, and treatment strategies remain under reported.

**Patient concerns:**

Here, we describe a 54-year-old Chinese man diagnosed with lung adenocarcinoma (cT4N3M1b, stage IV) with neuroendocrine differentiation and L858R mutation on exon 21. He developed progressive disease in liver 4 months later, and the biopsy of liver metastases showed neuroendocrine carcinoma maintained the same EGFR mutation.

**Diagnoses:**

Lung adenocarcinoma and neuroendocrine carcinoma were identified by biopsy.

**Interventions:**

After a combined treatment with nab-paclitaxel and erlotinib, the patient achieved partial remission.

**Outcomes:**

The patient's overall survival was 27 months.

**Lessons:**

This case highlights that EGFR mutated lung neuroendocrine carcinoma is not responsive to single-agent EGFR-TKI. However, combined application with nab-paclitaxel can improve its efficacy and prolong the patient's survival.

## 1 Introduction

**Figure 1:**
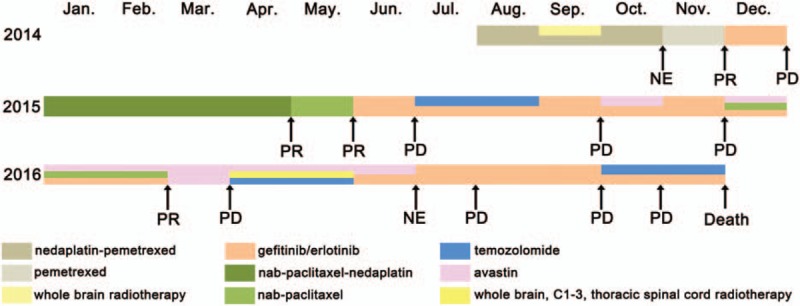
Patient's history of anticancer treatment. NE = not evaluated, PD = progressive disease, PR = partial remission.

Lung cancer is the leading cause of cancer-related death worldwide.^[1]^ During the past decade, identification of epidermal growth factor receptor (EGFR) mutation dramatically progressed the diagnosis and treatment of lung cancer. The rate of EGFR mutation in lung adenocarcinoma is reported to be 15% to 20%, and up to 44% to 51.4% in Asian populations.^[2–4]^ The identification of EGFR mutation has resulted in the development of tyrosine kinase inhibitor (TKI), which target mutated EGFR. EGFR-TKI has provided profound benefit to a group of EGFR mutation-positive lung adenocarcinoma patients. Treatment with EGFR-TKI has a significantly higher response rate and provides longer progression-free survival and better quality of life when compared with traditional platinum-doublet chemotherapy. EGFR mutation has rarely been reported in lung neuroendocrine carcinoma, which may affect the efficacy of TKI.^[5]^ Currently, patients with EGFR mutated lung neuroendocrine carcinoma are rare, and guidelines on the management of EGFR mutated lung neuroendocrine carcinoma are still under reported. Therefore, we describe a patient with lung neuroendocrine carcinoma with EGFR mutation, in which combined application of nab-paclitaxel and EGFR-TKI improves the efficacy of EGFR-TKI and prolongs the patient's survival (Fig. 1).

## 2 Case presentation

**Figure 2:**
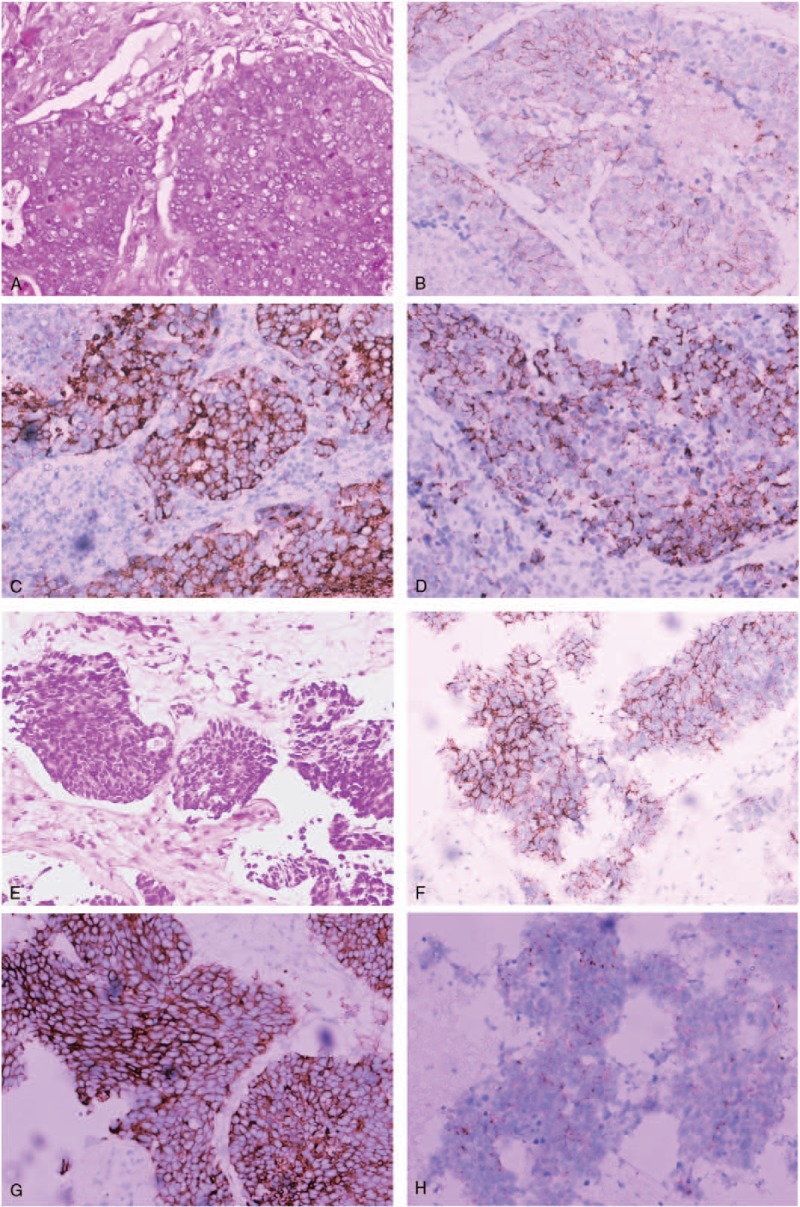
Biopsy of right supraclavicular lymph nodes and metastatic site in liver. (A) Hematoxylin–eosin staining of right supraclavicular lymph nodes shows acinar and papillary tumor cells. (B–D) The tumor cells in right supraclavicular lymph nodes were positive for CD56 (B), synaptophysin (C), and chromogranin (D) immunohistochemical staining. (E) Hematoxylin–eosin staining of metastatic site in liver show small-sized tumor cells with high nuclear to cytoplasmic ratio. (F–H) The tumor cells in liver were positive for CD56 (F), synaptophysin (G), and chromogranin (H). Magnification, ×200.

**Figure 3:**
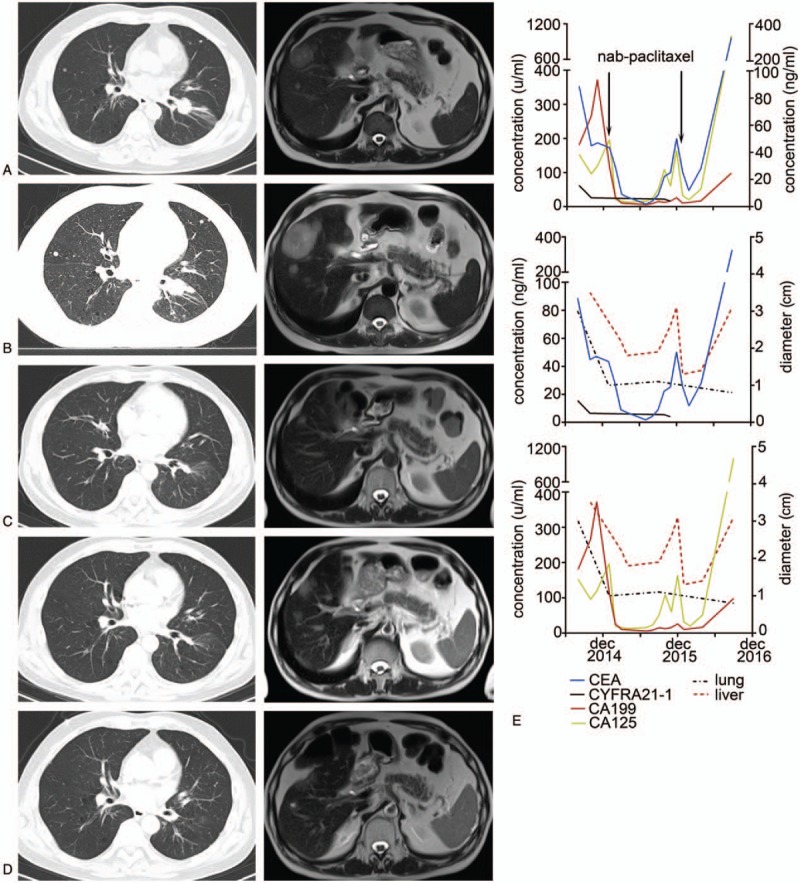
(A) After nedaplatin–pemetrexed chemotherapy, CT and magnetic resonance imaging in November 2014 before gefitinib treatment, implied lung cancer in left lower lobe and metastases in liver. (B) After treatment with gefitinib, lung masses on CT scan in January 2015 were stable, but metastases in liver progressed. (C) The patient achieved nearly complete remission in lung and partial remission in liver. (D) Upper panels: progression in liver in September 2015, lower panels: partial remission after treatment with paclitaxel–avastin and erlotinib, lesions in liver shrunk. (E) Dynamic changes of serum tumor markers including CEA, CYFRA21-1, CA199, and CA125, as well as the diameters of tumors in lung and liver are shown. CT = computed tomography.

A 54-year-old man (never smoker) was referred to our hospital in August 2014 because of nonproductive cough for 2 months and masses in the lung and liver identified with computed tomography (CT). Physical examination revealed a hard, fixed, and swollen right supraclavicular lymph node about 1.5 cm in diameter. The level of carcinoembryonic antigen (CEA) was 88.7 ng/mL. Positron emission tomography-CT (PET-CT) implied lung cancer (3.4 × 2.5 cm) in the left lower lobe with metastases in the lung, right supraclavicular lymph nodes, liver, and bones. Magnetic resonance imaging (MRI) of the brain revealed multiple abnormal signals in the cerebrum and cerebellum, implying multiple brain metastases. The patient was diagnosed with lung cancer (cT4N3M1b, stage IV). The subsequent biopsy of the right supraclavicular lymph node implied metastatic lung adenocarcinoma, which was morphologically an acinar and papillary pattern and was positive for CK7 and TTF-1 (Fig. 2A). Furthermore, the cancer cells showed neuroendocrine characteristics with positive immunohistochemical staining for CD56, synaptophysin and chromogranin, indicating neuroendocrine differentiation (Fig. 2B–D). EGFR mutation analysis showed a single point mutation L858R in exon 21. He received 4 cycles of nedaplatin–pemetrexed chemotherapy (nedaplatin 140 mg and pemetrexed 870 mg) and whole brain radiotherapy (36 Gy/12F), followed by 1 cycle of pemetrexed (850 mg) chemotherapy. Radiographic examination revealed a partial remission with 30% decrease in lung and liver lesions and complete regression in brain lesions (Fig. 3A). The level of CEA decreased to 44.8 ng/mL. Considering EGFR mutation status, he commenced gefitinib therapy (250 mg qd).

In January 2015, the patient had a mixed response to gefitinib therapy with stable lesions in lung but progressive metastases in liver (Fig. 3B). Biopsy of the liver metastases was obtained and disclosed metastatic poor-differentiated neuroendocrine carcinoma showing the same EGFR mutation as the first biopsy. The small-sized tumor cells had high nuclear to cytoplasmic ratio and were positive for CD56, synaptophysin, and chromogranin, implying neuroendocrine carcinoma (Fig. 2E–H). CK7 and TTF-1 were also positive (not shown). Considering that paclitaxel was reported to be effective in heavily pretreated or drug-resistant small cell lung cancer as well as nonsmall cell lung cancer (non-SCLC), we switched his treatment to paclitaxel-based chemotherapy. Four cycles of nab-paclitaxel–nedaplatin chemotherapy (nab-paclitaxel 400 mg and nedaplatin 130 mg) and 2 cycles of single-agent nab-paclitaxel chemotherapy (nab-paclitaxel 400 mg) were initiated and resulted in partial remission in lung and liver. CT scan demonstrated that lesions in lung and mediastinal lymph nodes almost completely regressed and lesions in liver shrunk about 70%. PET-CT also implied reduction of standard uptake value (SUV) in masses in lung (SUV = 1.8 vs 10.3 at initial diagnosis), in hilar, mediastinal, and cervical lymph nodes; in liver; and in thoracic vertebra T8, T10, and sternum. Then he restarted EGFR-TKI therapy with erlotinib (150 mg qd). A month later, the masses in the liver continued to shrink, but metastases in the brain progressed (Fig. 3C) although blood levels of CEA were normal. Because of disease progression in the brain, 2 cycles of temozolomide (200 mg qod, days 1, 3, 5, 7, 9), which was active and safe in brain metastases from tumors including lung cancer, was started. He then continued erlotinib treatment.

In September 2015, the level of CEA slightly increased to 8.79 ng/mL. CT scan and MRI revealed progression in liver and brain metastases. Four cycles of nab-paclitaxel (400 mg) plus avastin (400 mg) chemotherapy combined with erlotinib were administered, during which the level of CEA was reduced from 50.13 to 8.03 ng/mL. The patient experienced partial remission again, revealed by shrinkage of liver masses shown in MRI (Fig. 3D).

Despite erlotinib and nab-paclitaxel chemotherapy, the patient rapidly developed other symptoms including muscle weakness in both lower limbs in March 2016. MRI showed more metastases in the brain and new metastases in C2–3, T12-L1 spinal cord, and kidney. Masses in lung were stable and metastases in liver were smaller. The level of CEA was 28.14 ng/mL (Fig. 3E). After subsequent administration with 2 cycles of temozolomide–avastin chemotherapy (temozolomide 200 mg qd, days 1–5, avastin 400 mg, day 9) and concurrent radiotherapy of the whole brain, C1–C3 (18 Gy/6F) and thoracic spinal cord (42 Gy/14F), his muscle strength was improved. In addition, shrinkage in lesions in the cervical spinal cord was detected in the repeat MRI. Further, avastin and erlotinib treatment was performed as maintenance therapy. The patient survived for 27 months from the initial diagnosis until he died in November 2016.

## 3 Discussion

Current evidence-based guidelines for the management of advanced lung adenocarcinoma suggest analysis of EGFR mutation and recommend EGFR-TKI as first-line therapy for advanced lung adenocarcinoma with EGFR mutation on exons 19 and 21.^[6,7]^ However, the necessity to analyze EGFR mutation and to conduct EGFR-TKI treatment in other lung cancers is under reported. EGFR mutation in lung neuroendocrine carcinoma is extremely rare. Most are reported as transformation after TKI therapy or combination with adenocarcinoma.^[8–10]^ Among the reported lung neuroendocrine carcinomas harboring EGFR mutation, the rate in SCLC and large cell neuroendocrine lung cancer (LCNEC) is 1.8% to 4% and 0% to 3.7%, respectively.^[9,11–13]^ Those reported de novo SCLC with EGFR mutation were EGFR-TKI resistant.^[5,10]^ EGFR mutated SCLC transformed from or coexistent with adenocarcinoma also had poor response to EGFR-TKI and underwent rapid progression.^[14–16]^ The response of EGFR mutant LCNEC remains controversial.^[5,17]^ Therefore, most of the EGFR mutated lung neuroendocrine carcinomas, even with sensitizing EGFR mutation, may restrict the efficacy of EGFR-TKI. This may be explained by the lack of EGFR expression in neuroendocrine carcinoma. This histological distinction may exclude the function of EGFR as a driver oncogene.^[18]^ Because prognosis of advanced lung neuroendocrine carcinoma harboring sensitizing EGFR mutation is poor, defining strategies to manage these cases is an urgent problem for clinicians. However, there are no accepted guidelines on the management of these cases.

In our case, the patient was initially diagnosed was EGFR mutated adenocarcinoma with neuroendocrine differentiation, implying neuroendocrine features. The patient developed progressive disease in liver after gefitinib therapy for just 1 month and further biopsy in liver showed metastatic poor-differentiated neuroendocrine carcinoma. The distinction between the first and second biopsy suggested the necessity to conduct a repeat biopsy, which was essential for the treatment and prognosis prediction, especially when neuroendocrine features were identified previously. Despite EGFR mutation on exon 21, histological pathology of poor-differentiated neuroendocrine carcinoma and late clinical stage with multiple metastases in the lung, liver, and brain at the time of diagnosis meant a poor prognosis for this patient. Repeated progression of cancer in the liver and brain during EGFR-TKI therapy was consistent with this prognosis. Currently, the main treatment for advanced lung neuroendocrine carcinoma is chemotherapy.^[19,20]^ However, there are no accepted guidelines on whether to commence single-agent EGFR-TKI therapy or combined treatment with other chemotherapy. To our best knowledge, nab-paclitaxel–nedaplatin is a first-line chemotherapy regimen for advanced non-SCLC. It has been also proved to be effective in lung neuroendocrine carcinoma.^[21–23]^ After progression in the liver, the patient was given nab-paclitaxel plus erlotinib which eased the patient's symptom and led to partial remission. This regimen also demonstrated significant activity during the second progression and prolonged his survival.

In summary, we described a rare case of lung neuroendocrine carcinoma with EGFR mutation, which may predict the lack of response to EGFR-TKI and a poor prognosis. Combination treatment with nab-paclitaxel and EGFR-TKI may be more effective than single-agent EGFR-TKI.
