# Marked response to nab-paclitaxel in EGFR mutated lung neuroendocrine carcinoma: A case report: Erratum

**DOI:** 10.1097/MD.0000000000007644

**Published:** 2017-07-21

In the article, “Marked response to nab-paclitaxel in EGFR mutated lung neuroendocrine carcinoma: A case report”,^[1]^ which appeared in Volume 96, Issue 21 of *Medicine*, “Cancer Center, Union Hospital, Tongji Medical College, Huazhong University of Science and Technology, Wuhan, China” should have appeared as “Cancer Center, Union Hospital, Tongji Medical College, Huazhong University of Science and Technology, Wuhan 430022, China.”
